# P-1498. Contezolid is Active against Methicillin-Resistant *Staphylococcus aureus* in Experimental Rat Foreign Body Osteomyelitis

**DOI:** 10.1093/ofid/ofae631.1667

**Published:** 2025-01-29

**Authors:** Melissa J Karau, Sebastian Herren, Christina Koscianski, Kerryl Greenwood-Quaintance, Jay Mandrekar, Robin Patel

**Affiliations:** Mayo Clinic, Rochester, Minnesota; Mayo Clinic, Rochester, Minnesota; Mayo Clinic, Rochester, Minnesota; Mayo Clinic, Rochester, Minnesota; Mayo Clinic, Rochester, Minnesota; Mayo Clinic, Rochester, Minnesota

## Abstract

**Background:**

Methicillin-resistant *Staphylococcus aureus* (MRSA) is a common cause of orthopedic implant-associated infection, where *S. aureus* can survive intracellularly and form biofilms. Vancomycin has poor activity against staphyloccoccal biofilms. Although rifampin is effective against staphylococcoal biofilms and has intracellular activity, resistance may develop when it is used as a single agent. Contezolid is a novel oxazolidinone with reported activity against *S. aureus in vitro* and *in vivo* in both murine systemic and thigh infection models. Here, we assessed the *in vivo* activity of contezolid, with and without rifampin, in a rat model of foreign body osteomyelitis.

Figure 1.

**Figure:**
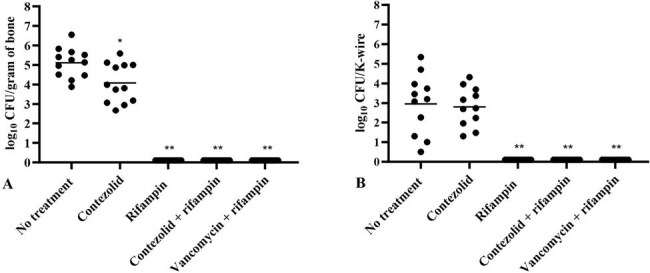

Quantities of MRSA IDRL-6169 recovered from A. tibia B. K-wire. Circles represent values from individual animals; horizontal lines represent mean values. Compared to no treatment: *p=0.0186 **p<0.0002

**Methods:**

The left tibia of 60 male Wister rats were inoculated with arachidonic acid (sclerosing agent) and 10^6^ cfu MRSA IDRL-6169 and a K-wire implanted. After four weeks, rats received either no treatment, contezolid (50 mg/kg orally, every 12 h), rifampin (10 mg/kg intraperitoneally every 12 h), rifampin plus contezolid, or vancomycin (75 mg/kg intraperitoneally, every 12 h) plus rifampin for 21 days. Following treatment, tibiae were harvested and cyropulverized. Tibiae and K-wires were cultured separately, and results reported as log_10_ cfu/gram of bone or K-wire (limit of detect 0.1 log_10_), respectively. Wilcoxon rank-sum test and false discovery rates were performed and p< 0.05 considered significant.

**Results:**

Results are shown in Figure 1. MRSA was recovered from tibiae and K-wires of all untreated animals, at means of 5.1 log_10_ cfu/bone and 3.0 log_10_ cfu/K-wire, respectively. There was a 1.0 log_10_ cfu/g mean reduction in MRSA recovered from the tibiae of contezolid-treated rats compared to untreated rats (p=0.0186), and a 0.2 log_10_ reduction on K-wires. No MRSA was recovered from the tibiae or K-wires in animals receiving rifampin, whether alone or in combination with contezolid or vancomycin.

**Conclusion:**

Contezolid was active against MRSA in a rat model of foreign body osteomyelitis, reducing bacterial loads in bone, albeit not on K-wires, unless co-administered with rifampin.

**Disclosures:**

**Robin Patel, MD**, a patent on Bordetella pertussis/parapertussis PCR issued, a patent on a device/method for sonication with royalties paid by Samsung to Mayo Clinic, a: See above|MicuRx Pharmaceuticals and BIOFIRE: Grant/Research Support|PhAST, Day Zero Diagnostics, Abbott Laboratories, Sysmex, DEEPULL DIAGNOSTICS, S.L., Netflix, Oxford Nanopore Technologies and CARB-X: Advisor/Consultant|Up-to-Date and the Infectious Diseases Board Review Course.: Honoraria

